# The influence of pressure on crude oil biodegradation in shallow and deep Gulf of Mexico sediments

**DOI:** 10.1371/journal.pone.0199784

**Published:** 2018-07-03

**Authors:** Uyen T. Nguyen, Sara A. Lincoln, Ana Gabriela Valladares Juárez, Martina Schedler, Jennifer L. Macalady, Rudolf Müller, Katherine H. Freeman

**Affiliations:** 1 Department of Geosciences, The Pennsylvania State University, University Park, Pennsylvania, United States of America; 2 Institute of Technical Biocatalysis, Hamburg University of Technology, Hamburg, Germany; National Sun Yat-sen University, TAIWAN

## Abstract

A significant portion of oil released during the Deepwater Horizon disaster reached the Gulf of Mexico (GOM) seafloor. Predicting the long-term fate of this oil is hindered by a lack of data about the combined influences of pressure, temperature, and sediment composition on microbial hydrocarbon remineralization in deep-sea sediments. To investigate crude oil biodegradation by native GOM microbial communities, we incubated core-top sediments from 13 GOM sites at water depths from 60–1500 m with crude oil under simulated aerobic seafloor conditions. Biodegradation occurred in all samples and followed a predictable compound class sequence dictated by molecular weight and structure. 45 to ~100% of total *n-*alkane and 3 to 60% of total polycyclic aromatic hydrocarbons (PAH) were depleted. In reactors incubated at 4°C and at pressures of 6–15 MPa, the depletion in total *n*-alkane was inversely correlated to pressure (R^2^ ~ 0.85), equivalent to a 4% decrease in total *n*-alkane depletion for every 1 MPa increase. Our results indicated a modest inhibitory effect of pressure on biodegradation over our experimental range. However, the expansion of oil exploration to deeper waters (e.g., 5000 m) opens the risk of spills at conditions at which pressure might have a more pronounced effect.

## Introduction

The 2010 Deepwater Horizon (DWH) blowout created the first major oil spill in deep waters. It released ~5 million barrels of Macondo oil to the Gulf of Mexico (GOM) at a water depth of 1500 m. An estimated 3–31% of the oil was transported to the seafloor, contaminating a region of 3200 km^2^ around the Macondo wellhead [1, 2]. Oil sedimentation was promoted by marine oil snow formation and flocculent accumulation (“MOSSFA”) [3, 4] which created oil-particle aggregates able to sink from surface waters or from the deep intrusion layers that formed in the water column at depths of 1000–1300 m [5]. These subsurface oil plumes, rather than oil that reached surface waters, were considered a major source of oil to the seafloor, based on evidence of minimal photodegradation in oiled sediment samples [2]. Sinking high-density oil residues [6] and diffusion through the water column [7] may also have contributed to oil sedimentation.

Little information about the fate of oil spilled in deep-sea environments was available before the Deepwater Horizon blowout, and it was unclear how much could be extrapolated from studies of previous spills in very different environments (e.g. Exxon Valdez [8] and Gulf War [9]). Biodegradation is expected to be the major depletion mechanism of oil in deep, dark waters [10], where other common weathering processes in surface waters such as photooxidation and evaporation are not active. This expectation was reinforced by studies that revealed the enrichment of indigenous oil-degrading microbes and upregulation of hydrocarbon-degrading genes in deep waters following the spill [11–14]. Additionally, Stout & Payne [15] and Bagby et al. [16] found a significant depletion in various Macondo compound classes in deep (1000–1912 m) GOM sediments over the 4 years following the spill, indicating that indigenous microbial communities of the deep sea actively degrade oil components.

Deep sea environments, characterized by low temperature and high hydrostatic pressure, present energetic challenges to microbial metabolism. Among interconnected factors (e.g., physical conditions, nutrient and oxygen levels, background organic matter, and microbial community composition) that likely control hydrocarbon biodegradation on the seafloor [17, 18], the influence of pressure is least studied. Laboratory incubation experiments [19–25] have demonstrated that some bacteria are capable of hydrocarbon degradation under elevated pressure, but the effect of pressure in these studies has been mixed. Schwarz et al. [19, 20] discovered a 10 x decrease in rates of growth and hexadecane utilization of a microbial culture isolated from 4940-meter-deep sediments in the Atlantic Ocean at 50 MPa compared to the same culture incubated at ambient pressure (0.1 MPa). Grossi et al. [23] conversely, found no inhibitory effect of pressure on the growth and hexadecane consumption of piezotolerant, alkane-degrading *Marinobacter hydrocarbonoclasticus* strain #5 at 35 MPa. While 15 MPa slightly inhibited *Rhodococcus qingshengii TUHH-12* growth on *n*-hexadecane, it completely halted *Sphingobium yanoikuyae B1* growth on naphthalene [25]. In the first experimental study of the effect of pressure on oil degradation using environmental samples containing mixed microbial assemblages, Prince, Nash, and Hill [26] found that crude oil biodegradation by a surface water inoculum was 33% slower at 15 MPa than at surface pressure (0.1 MPa).

The expansion of oil exploration and production to deeper marine environments increases the likelihood of deep-sea oil spills. However, laboratory studies of the effect of pressure on hydrocarbon biodegradation have only focused on the fate of individual oil model compounds (e.g., hexadecane and naphthalene) or of crude oil in the water column. Biodegradation occurring in the water column, however, might not represent that in sediments, owing to potential differences between the two systems such as microbial concentration and access to hydrocarbon substrates. In this work, we investigated the rate and extent of crude oil biodegradation in sediments from the Northern GOM, collected at water depths from 62–1520 m, with a specific focus on the role of pressure. We approximated in-situ temperatures and pressures of sediments in 18-day incubation experiments with crude oil and examined changes in gas chromatography (GC)—amenable hydrocarbons. This is the first comparative study of crude oil biodegradation by indigenous microbes in sediments under deep and shallow marine conditions, designed to assess the potential for natural attenuation of spilled oil in GOM sediments.

## Materials and methods

### Incubation experiments

**Fig 1:**
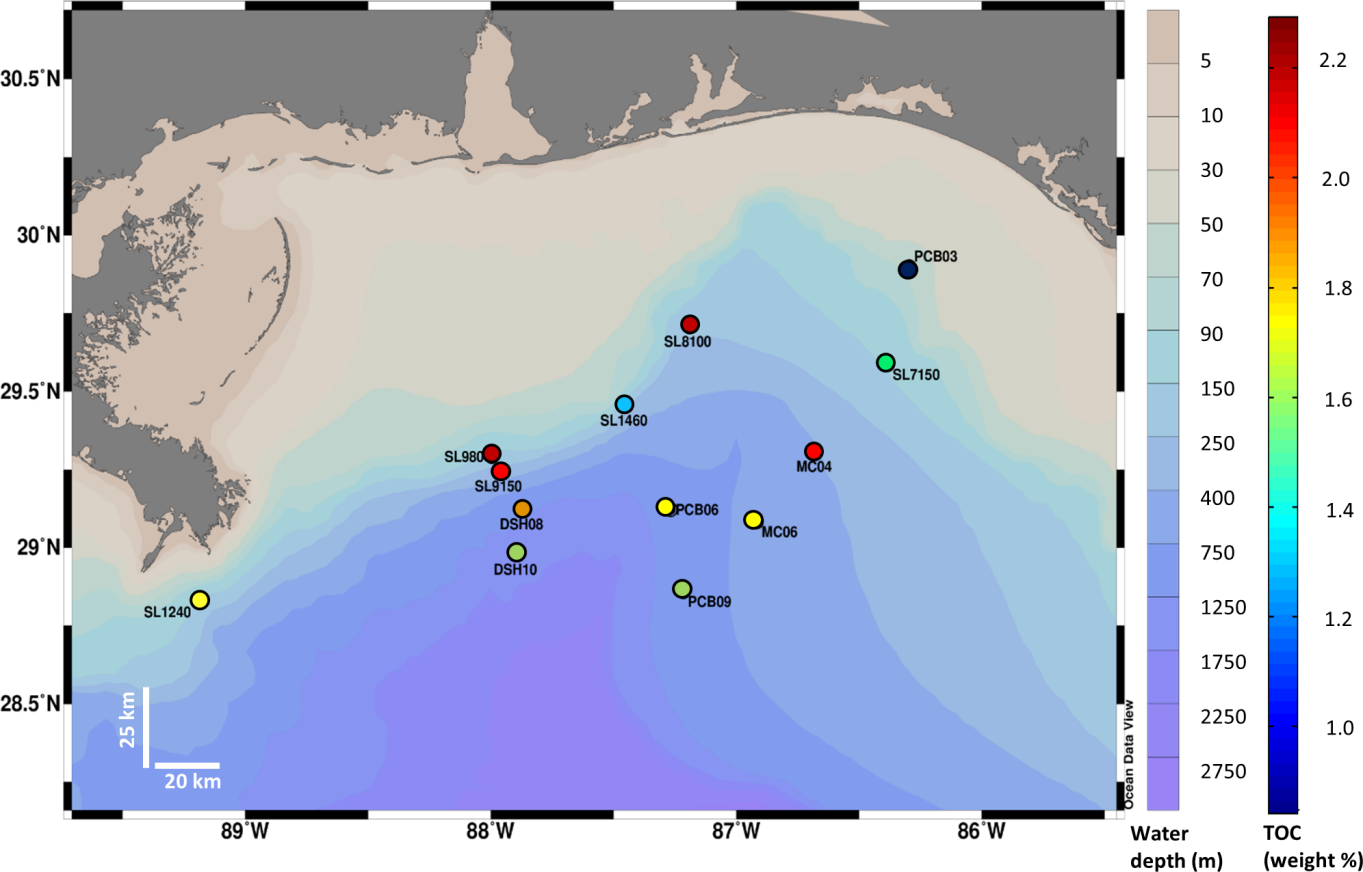
Map of sampling sites. Locations of 13 sampling sites across the Northern Gulf of Mexico at water depth ranging from 60–1520m. Circles are color-coded representing total organic content (TOC, percent weight of sediments). Schlitzer, R., Ocean Data View, http://odv.awi.de, 2016.

Thirteen sediment cores were collected in the Northern Gulf of Mexico (GOM) at water depths ranging from 62 to 1520 m, using a multicorer (Ocean Instruments MC-800) deployed from the R/V WeatherBird II ship, in August 2014. Sampling area spanned from 28°49’36” N to 29°53’56” N and from 86°17’40” W to 89°30’48” W (Fig 1, Table 1). Field area was not on any private land, no permissions were required for collecting sediment cores at these sites and this study did not involve endangered or protected species. Approximately 0.2 g of coretop (0–4 mm) sediment from each site were amended with 5 μL autoclaved sweet Louisiana crude, a Macondo oil surrogate, and 5 mL of minimal mineral medium following DSMZ methanogenium medium 141 recipe [25, 27] and vortexed. Incubation conditions approximated in-situ physical environments of the sediments: pressure ranged from 0.1 to 15 MPa and temperatures were 4, 10, and 20°C. For each sediment site, we incubated oil-amended sediment in duplicate, with a parallel control of un-amended sediment. An oil-amended control was frozen to -20°C immediately after shaking and was used to determine the initial extractable oil composition. Sediments were incubated at pressures ranging from 0.1 to 15.3 MPa, selected in order to approximate *in situ* pressures for the sample (Table 1). Incubation vials in > 0.1 MPa experiments were placed in stainless steel reactors that were capped with bronze lids and pressurized with nitrogen gas [28]. Incubation vials in ambient pressure experiments (0.1 MPa) were placed in equivalent aluminum reactors. In addition, to further explore the effects of pressure, three deep sediment samples were incubated both at high pressures (9.4, 11.1, and 15.3 MPa) and at 0.1 MPa and 4°C. Because core-top sediments were relatively well-oxygenated *in situ* (Table 1), all experiments were carried out under aerobic conditions. Incubation vials were stirred at 200 rpm with magnets to keep oxygen, sediments, and nutrients well-mixed over the course of the incubation period. Experiments were stopped after 18 days and frozen at -20°C until analysis.

**Table 1:** Physical conditions of sediment sites and laboratory incubation conditions. (CTD: conductivity-temperature-depth device, P: pressure, T: temperature).

Site	Water depth (m)	Latitude	Longitude	CTD bottom water T (°C)	CTD bottom water oxygen (μmol/kg)	Incubation P initial (MPa)	Incubation P final (MPa)	Incubation T (°C)	Comments
SL1240	62	28 49.592	89 30.796	20.71	129.26	0.1	0.1	20	**Incubation conditions approximated in situ pressure and temperature conditions**
PCB03	96	29 53.935	86 17.68	18.33	126.77	0.1	0.1	20	
SL980	150	29 17.513	88 02.512	16.08	118.28	2.5	2.5	20	
SL7150	196	29 35.231	86 22.84	10.91	111.6	1.9	1.8	10	
SL1460	212	29 27.348	87 26.994	18.28	126.69	2.5	2.1	20	
SL8100	226	29 42.428	87 11.338	11.04	109.52	1.9	1.9	10	
SL9150	251	29 14.957	87 59.750	11.12	118.76	2.5	2.3	10	
MC04	399	29 18.44	86 40.495	9.42	112.55	4.0	3.6	10	
MC06	595	29 5.013	86 54.871	7.26	124.08	5.8	5.4	4	
PCB09	981	28 51.548	87 12.888	5.22	165.33	10.5	9.4	4	
PCB06	1008	29 07.371	87 15.928	5.18	165.86	9.4	8.7	4	
DSH08	1127	29 07.378	87 52.091	4.83	176.5	11.1	10.5	4	
DSH10	1520	28 58.687	87 53.438	5.07	169.4	15.28	14.08	4	
**PCB06**	1008	29 07.371	87 15.928	5.18	165.86	0.1	0.1	4	**Ambient pressure replicates of the three deepest sites**
**DSH08**	1127	29 07.378	87 52.091	4.83	176.5	0.1	0.1	4	
**DSH10**	1520	28 58.687	87 53.438	5.07	169.4	0.1	0.1	4	

### Organic extraction and analysis

Total organic content (TOC) of core-top sediments was measured as weight percent carbon of sediment using a Leco C/S-744 analyzer after sediments were treated with hydrochloric acid 1N to remove inorganic carbon. Incubation vials were centrifuged to separate aqueous and solid phases in order to measure the water fraction and sediment-associated oil components. Any visible oil on vial walls after decanting was recovered with additional sea water medium and transferred to the water fraction (WAF). For each sample, both phases were extracted with an azeotrope of dichloromethane and methanol (in a proportion of 9:1 by volume) three times. Liquid phases (~5 mL) were extracted with a total of 15 mL, while sediments (~ 0.2 g) were extracted with a total volume of 10 mL solvent. Organic extracts were separated into aliphatic, aromatic, and polar fractions by silica gel chromatography using 100% *n*-hexane, *n*-hexane and dichloromethane (4:1, v/v), and dichloromethane and methanol (4:1, v/v)), respectively, as eluents (S1 Fig). The aliphatic and aromatic fractions, represented in the first and second eluted fractions, were analyzed on a Trace 1310 gas chromatography (GC) coupled to an ISQ LT single quad mass spectrometer (MS) (Thermo Scientific) (S1 Appendix). Polycyclic aromatic hydrocarbons (PAHs) in the aromatic fraction were further characterized on an Agilent HP 6890 GC coupled to a HP 5973 mass selective detector in selected ion monitoring (SIM) mode due to the higher peak resolution on this system (S1 and S2 Tables). *N*-alkane and branched alkanes were quantified using an alkane standard mix of C_7_-C_40_ solution (Sigma-Aldrich). Parent PAHs and their alkylated homologues were quantified using a standard mix of 16 EPA priority PAH (Sigma-Aldrich) (S1 Appendix).

### Biodegradation parameters

To characterize and quantify biodegradation effects on oil components, we normalized compounds to internal biomarkers generally considered to be recalcitrant [29–31]. Aliphatic compounds were normalized to 17α(H),21β(H)-hopane (C_30_ hopane, detected and quantified with *m/z* 191) and aromatic compounds were normalized to C_26_ triaromatic sterane (C_26_ TAS, detected and quantified with *m/z* 231), both of which were abundant in the amended oil. The relative loss of different compound classes was calculated as following (t_0_ and t_f_ are the initial and final time points for the incubation):

Totaln-alkaneloss(%)=[1−[∑n−alkaneC30hopane]tf[∑n−alkaneC30hopane]t0]*100 (1)

TotalPAHloss(%)=[1−[∑PAHC26TAS]tf[∑PAHC26TAS]t0]*100 (2)

We defined total *n*-alkanes as the sum of C_15–40_ *n*-alkanes and total PAH as the sum of all PAHs analyzed (S1 Table). We also determined ratios of biomarker abundances that are commonly used in petroleum biodegradation studies such as C_17_ *n*-alkane/pristane, C_18_ *n*-alkane/phytane, ∑C_15–20_ *n*-akane/∑C_15–40_ *n*-alkane, and isomer ratios of mono-methylated PAH [32].

## Results and discussion

### Biodegradation sequence

**Fig 2:**
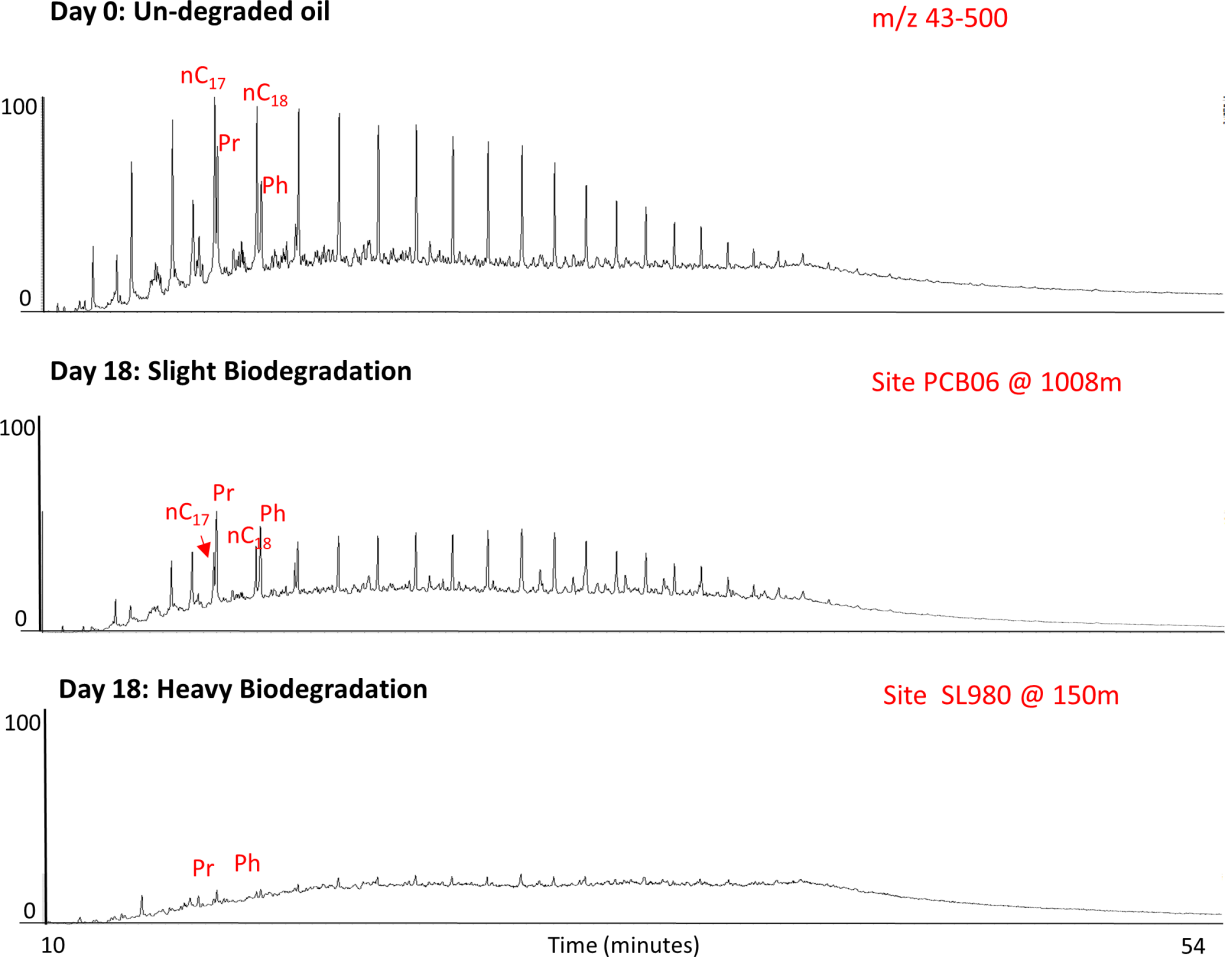
Chromatograms of crude oil biodegradation. Examples of total ion chromatograms of oil extract (normalized to C_30_ hopane) from two extremes of biodegradation at different water depths (nC17 = C17 *n*-alkane, nC18 = C18 *n*-alkane, Pr = Pristane, Phy = Phytane).

Compound loss patterns after incubation followed the canonical biodegradation sequence [31–34] and were consistent with field data on Macondo oil degradation [15, 16]. The loss sequence was governed by molecular weights and structures; short chain alkanes were degraded to a greater extent than long chain alkanes (S2 Fig), and straight chain *n*-alkanes were preferentially degraded over their saturated isoprenoid analogues (Fig 2). Long chain *n*-alkanes up to C_40_ were degraded, suggesting that these long alkanes were more susceptible to biodegradation than C_30_ hopane; these results contrast with those reported by Bagby et al., who used C_40_ *n*-alkane as conservative tracer due to its recalcitrance in biodegradation [16]. Total PAH decreased to a smaller extent than total n-alkanes, with the resistance to biodegradation increased with the number of rings and the degree of alkylation. For instance, 3-ring PAHs including phenanthrene and its alkylated homologues were depleted in most samples, whereas 4-ring PAHs such as pyrene and chrysene were only slightly degraded in the most degraded samples (S3 Fig).

We used C_30_ hopane and C_26_ TAS as conservative oil biomarkers in our analyses. Compound groups such as hopanes, steranes, and TAS have been widely used as conservative tracers for oil, based on the assumption that they are relatively recalcitrant [29–31]. However, recent laboratory studies [35–37] and field data [15, 16] have shown that these compounds can be more subject to biodegradation than previously thought. We justified the treatment of C_30_ hopane and C_26_ TAS as conservative tracers in our study for two reasons. First, our incubation duration (18 days) was shorter than the time scales of hopane and sterane biodegradation observed in the field [38] and experimentally. Homohopane biodegradation in laboratory experiments was reported to begin after 3–5 weeks at 30°C [35–36], while no degradation of TAS occurred over 21 days of oil incubation at 37°C [39]. Second, our experiments showed no change in ratios of R/S isomers of homohopane series (S2 Appendix), as is usually observed during biodegradation of these biomarkers [40–43].

### Alkane degradation and pressure inhibitory effect

**Fig 3:**
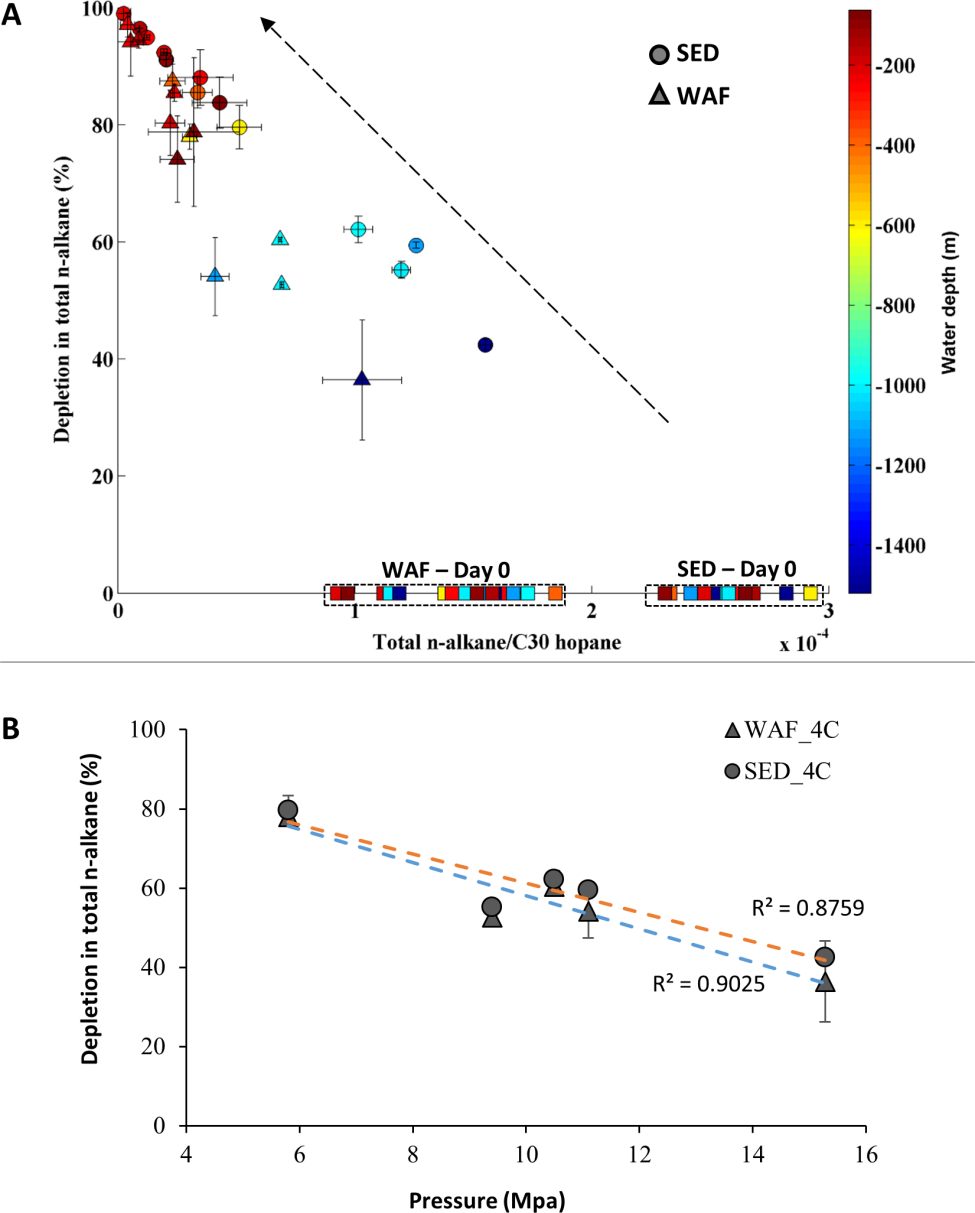
Total n-alkane degradation. Depletion of total *n*-alkane (%) after 18 days of incubation in both water fraction (WAF, triangles) and sediment fraction (SED, circles), the dashed arrow is interpreted as the direction of increasing biodegradation extent: A, All samples: Initial total *n*-alkanes are represented by squares. Samples are color-coded according to sampling water depths and B, Inhibitory effect of pressure on n-alkane biodegradation at 4°C. Error bars represent one standard deviations from the means.

After 18 days, total *n*-alkanes were depleted in all samples. The percent loss of total alkanes ranged from 40% to 100%, and samples incubated at lower pressures (< 5 MPa) had more than 80% alkane depletion. Replicate incubations exhibited a small range of variability, with standard deviations from 0.03 to 5% (S3 Table). The extent of biodegradation was greater at shallower sites than at deeper sites (p < 0.05, one tailed *t*-test, Figs 2 and 3A, Table 2). Degradation of oil in both sediment and water fractions were relatively similar at each site. For all samples incubated at higher pressures (i.e., from 5.8 to 15 MPa, at 4°C), total *n*-alkane loss was inversely proportional to pressure (r^2^ > 0.85). This linear relationship represents ~ 4% decrease in the rate of alkane loss via biodegradation per 1 MPa increase, assuming simple first order kinetics (Fig 3B). The rate of *n*-alkane loss was slowest in samples incubated at 15 MPa, and ~ 36% less than in their counterparts incubated at 0.1 MPa and ~ 55% slower than samples incubated at 0.1 to 2.5 MPa from other sediment sites.

**Table 2:** Comparison in biodegradation of *n*-alkanes and PAHs between shallow (< 500 m) and deep (> 500 m) sediments.

Group by depth	*n*-alkane–SED (%)	*n-*alkane–WAF (%)	PAH–SED (%)	PAH–WAF (%)
< 500 m	91.4 ± 5.1	73.6 ± 14.6	33.5 ± 25	29.8 ± 15.8
> 500 m	62.4 ± 12.5	45.6 ± 17.4	34.5 ± 19.9	27.4 ± 9.1
*t*-test p value	0.00007	0.001	0.92	0.64

Mean ± one standard deviation of depletion percent in total *n-*alkane and total PAH, and p values for one tailed *t*-tests with significant level α = 0.05 (SED: sediment fraction, WAF: water fraction).

We calculated mean half-lives (assuming a first-order rate law) for total *n-*alkane to be ~ 21 days at 15.3 MPa, and ~ 9 days at 0.1–2.5 MPa. Our results are consistent with those of Prince, Nash, and Hill [26] who observed a 33% reduction in degradation rate at 15 MPa compared to 0.1 MPa, using a water column inoculum amended with 3 ppm oil. We also found inverse relationships between loss via biodegradation and water depth for other aliphatic compounds, including cyclohexanes, pristane, and phytane (S4 and S5 Figs).

### PAH degradation

**Fig 4:**
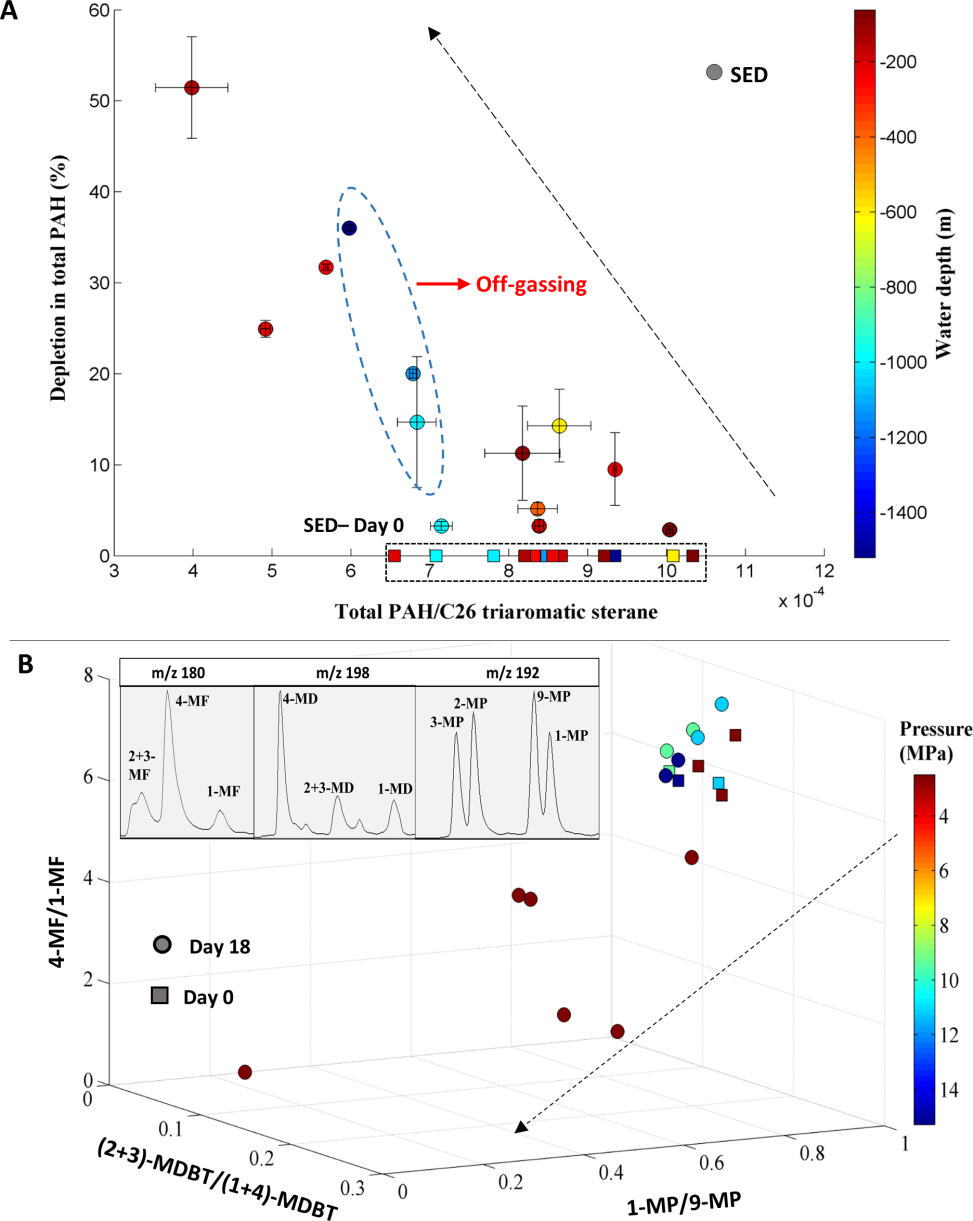
PAH degradation. Depletion of total PAH (%) of crude oil in sediment fraction (SED, circles) at after 18 days of incubation. The dashed arrow is interpreted as the direction of increasing biodegradation extent. A, All samples: Initial total PAHs are represented by squares. Samples are color-coded according to sampling water depths. Depletion in deep water samples are possibly due to off-gassing effect. B, Distinguishing biodegradation from off-gassing, using different isomer ratios of methylated-PAHs (MF: methyl fluorene *m/z* 180; MD: methyldibenzothiophene *m/z* 198; MP: methylphenanthrene *m/z* 192). Samples are color coded by pressures (MPa). Error bars represent one standard deviations from the means.

Overall, total PAH concentrations decreased as much as 60% after incubations, and standard deviations averaged 19% between replicates from each site (S3 Table). There was no significant difference between shallow and deep sediments (p > 0.05, one tailed *t*-test, Table 2). Sediments from the shallowest water depths (incubated at 0.1 MPa) only exhibited limited PAH biodegradation. Samples incubated at 2.5 MPa showed the greatest extent of PAH depletion, consistent with having the greatest *n*-alkanes degradation. High pressure samples (9.4–15 MPa) also showed decreases in total PAHs, though to a smaller extent than at 2.5MPa. Depletion of total PAHs at 15 MPa (~ 35%) was comparable to PAH depletion in samples incubated at 2.5 MPa. This was surprising since the 15 MPa sample showed the least n-alkane depletion. This led us to consider the potential for an experimental artifact due to loss of volatile compounds during sample decompression following the incubation period. Indeed, when this is accounted for, we observed a trend toward greater PAH loss at lower pressures (Fig 4A and S6 Fig). To estimate the effect of off-gassing, we used ratios of methylated homologues of phenanthrene (MP), fluorene (MF), and dibenzothiophene (MDBT). Biodegradation of hydrocarbons is often isomer-specific. Isomers may share similar physicochemical properties yet be more or less susceptible to biodegradation [34, 44–46], possibly due to enzyme specificity or steric considerations. At low pressures (2.5 MPa), samples with high levels of preferential degradation of certain methylated PAH isomers was consistent with previous published studies. For instance, we detected a decrease in the ratio of 1-MP/9-MP in degraded samples at 2.5 MPa, which is consistent with 9-methylphenanthrene (9-MP) being the most resistant to microbial oxidation among all MP isomers [47]. In contrast, this ratio remained relatively constant in 15 MPa samples, indicating both compounds, which have similar vapor pressures, were loss during de-gassing to the same extent. Similar consistency of ratios was observed for (2-MDBT+3-MDBT)/(1-MDBT+4-MDBT) and 4-MF/1-MF (Fig 4B). Attributing all PAH loss at 15 MPa (~ 35%) to off-gassing, we calculated that off-gassing only accounted for a maximum of ~ 3.5% loss in total n-alkane depletion at 15 MPa (out of a total loss of ~ 42%) (S3 Appendix). Thus, we concluded that biodegradation was indeed the major cause for n-alkane depletion at 15 MPa.

### Factors controlling biodegradation

Even before anthropogenic influence, the GOM seafloor was subject to petroleum input via natural seeps (average of 140,000 tons of petroleum annually) [48], which have likely been active over millions of years. Continued exposure may have primed GOM microbial communities to develop the capability to readily degrade hydrocarbons. Prior exposure to hydrocarbons could accelerate biodegradation, as a memory response [49]. We speculated that our sediments were previously exposed to oil, based on the presence of background oil hydrocarbons including *n*-alkanes and C_30_ hopane (S4 Appendix). In fact, several sites are within the area impacted by Macondo oil, including the three deepest water sites (DSH08, DSH10, and PCB06) [2, 16, 50]. This might explain the promptness in degrading oil of the GOM sediments seen in our study.

The level of hydrocarbon contamination in sediments has been proposed to influence rates of biodegradation [16, 51, 52]. In our study, oil amendment led to an average concentration of 1.1 μg C_30_-hopane/g sediment (S5 Appendix). This equates to a state of “heavy oil contamination” as defined by Valentine et al. (2014), who used a threshold of >750 ng/g in GOM sediments [2]. Samples at 2.5 MPa showed extensive biodegradation (~100% ∑n-alkanes, ~60% ∑PAHs depletion after 18 days) despite having similar heavy contamination level as deep sites, suggesting that contamination level was not a direct inhibitory factor, and that other factors such as nutrient and oxygen concentration, and microbial community composition might be more important rate-limiting forces.

There are inevitable challenges in isolating the effect of pressure on biodegradation. In previous studies of pressure effects, single inocula were incubated under both high and low pressure; either sea surface inocula were introduced to high pressure [26] or piezotolerant strains were placed in ambient pressure [19, 20]. Introducing microbes to non-native conditions can impact their growth and carbon utilization [53–55]. In this study, we attempted to minimize this concern by comparing the hydrocarbon-degrading capacity of native sediment communities under approximated in-situ conditions (although our sediments were exposed to surface conditions for a period after sampling). Given possible compromising factors deep-sea microbial communities encountered during sampling and experimental setup, we recognize that our results may provide a conservative estimation of biodegradation at high pressure.

To better understand the impact incubation under non-native conditions might have, we incubated three deep GOM sediments at both in situ seafloor (9.4–15 MPa) and atmospheric pressure. Hydrocarbon degradation in these high and low pressure treatments of the same sediments appeared to be stochastic. The DSH10 sample showed more extensive n-alkane biodegradation at surface pressure (0.1 MPa) than at seafloor pressure (15 MPa), consistent with an inhibitory effect of pressure. Conversely, the DSH08 sediment showed much less n-alkane degradation at surface pressure than at seafloor pressure (11 MPa). The PCB06 sample, however, showed virtually no difference in biodegradation between surface and seafloor pressure (9.4 MPa) treatments (Table 3). The absence of a clear trend in this subset of our data may be the result of pressure-induced perturbation in sediment community. We conclude that, until technology for in-situ deep sea incubation [56, 57] or pressure-retaining sampling [58, 59] becomes more widely available, the best practice for hydrocarbon biodegradation studies is to incubate samples under conditions simulating their native, in-situ environments.

**Table 3:** Comparison of total *n*-alkane depletion percent (mean ± one standard deviation) between high in-situ pressure incubation of the three deepest sites and their ambient pressure (0.1Mpa) incubation counterparts.

Sample	In-situ water depth (m)	Total *n*-alkane depletion (%)	
		In-situ Pressure	Surface pressure
DSH10	1520	42.5 ± 0.92	66.5 ± 0.57
DSH08	1127	59.5 ± 0.55	12.05 ± 6.5
PCB06	1008	55.2 ± 1.44	46.9 ± 3

## Conclusions

Our study assessed the rate and nature of oil biodegradation across the Northern GOM at a wide water depth range (60–1520 m), representing a range of shallow water to approximately the depth of DWH spill. All sediments were found to degrade oil. Piezotolerant microbial cultures at pressure up to 15 MPa demonstrated their capability to degrade oil, suggesting a high potential for natural attenuation of spilled oil. Under optimal nutrients and oxygen availability, as provided here, we predict that it would take a minimum of 42 days for complete n-alkane degradation at 15 MPa, compared to average of 19 days at shallow sites (0.1–2.5 MPa), assuming first order kinetics. Our study focused on the early, oxic biodegradation of GC-amenable oil, after 18 days of incubation. However, we expect that if the experiments were left to run longer on the scale of months or years with sufficient oxygen and nutrient supply, biodegradation could extend to other compound classes such as >4-ring PAHs and biomarkers (e.g., hopanes, steranes). Although pressure alone was not a major inhibitor of biodegradation in our experimental range, the expansion of oil exploration to deeper waters (e.g., 5000 m) opens the risk of spills at conditions at which pressure might have a more significant effect.

## Supporting information

S1 Appendix Gas chromatography–Mass spectrometry (GC-MS) and quantification methods. (DOCX)

S2 Appendix Distribution of hopanes and triaromatic sterane compound groups, showing the similarity between day 0 and day 18 samples, to justify the use of C30 hopane and C26-TAS as internal conservative oil biomarker in our study. (DOCX)

S3 Appendix Calculation of *n*-alkane depletion due to off-gassing at 15 Mpa. (DOCX)

S4 Appendix Background hydrocarbons in un-incubated sediments. (DOCX)

S5 Appendix Calculating contamination level. (DOCX)

S1 Table Oil hydrocarbons analyzed in this study and their quantitative molecular ion (*m/z)*. (DOCX)

S2 Table Selected Ion Monitoring method for alkylated PAHs. Each compound group is identified based on a quantitative ion and a confirmation ion *m/z* (Zeigler et al., 2008; Robbat Jr. and Wilton, 2014*). * Zeigler C., MacNamara K., Wang Z., Robbat Jr. A. Total alkylated polycyclic aromatic hydrocarbon characterization and quantitative comparison of selected ion monitoring versus full scan gas chromatography/mass spectrometry based on spectral deconvolution. Journal of Chromatography A 2008; 1205, 109–116. Robbat Jr., A.; Wilton, N.M. A new spectral deconvolution–Selected ion monitoring method for the analysis of alkylated polycyclic aromatic hydrocarbons in complex mixtures. Talanta 2014 125, 114–124.
(DOCX)

S3 Table Depletion (%) of total n-alkane and total PAH of individual samples. (TOC: total organic carbon, Carb: carbonate in the sediments, P: pressure, T: temperature, WD: water depth, SED: sediment fraction, WAF: water fraction).
(DOCX)

S1 Fig Summary of experimental and analytical procedures. (TIF)

S2 Fig Depletion of different *n*-alkanes to compare the extent of biodegradation as number of carbon increases. A. Mean and standard errors plot for depletion of each *n*-alkane for all day-18 samples; B. Boxplot for depletion of each n-alkane for all day-18 samples.
(TIFF)

S3 Fig Depletion of different PAH compound groups to compare the extent of biodegradation as number of rings increases. (C1: methyl, C2: ethyl or dimethyl, C3: trimethyl, C4: tetramethyl; Naph: napthalene, PNT: phenanthrene, Fluo: fluorene, DBT: dibenzothiophene, Py: pyrene, 11H-benzoF: 11H-benzo[b]fluorene, Chy: chrysene).
(TIFF)

S4 Fig Depletion in total cyclohexanes (m/z 83) after 18 days. The dashed arrow represents interpreted direction of increasing biodegradation extent.
(TIF)

S5 Fig Change in ratios of C_17_ n-alkane/pristane and C_18_ n-alkane/phytane after 18 days. A, sediment fractions (SED) and B, water fractions (WAF). Initial ratios are represented by the black square. Samples are color-coded according to sampling water depths. The dashed arrow represents interpreted direction of increasing biodegradation extent.
(TIF)

S6 Fig Depletion of total PAHs after 18 days of incubation of oil in water fraction (WAF, triangles). Initial total PAHs are represented by squares. Samples are color-coded according to sampling water depths. The dashed arrow represents interpreted direction of increasing biodegradation extent.
(TIF)
