# Supplementary material for: The influence of pressure on crude oil biodegradation in shallow and deep Gulf of Mexico sediments
Source: PLoS One. 2018 Jul 3;13(7):e0199784. doi: 10.1371/journal.pone.0199784 (PMC6029805; doi:10.1371/journal.pone.0199784)
Supplement: S1 Appendix — (DOCX) [file pone.0199784.s001.docx]

**S1 Appendix. Gas chromatography – Mass spectrometry (GC-MS) and quantification methods**

**Trace 1310 gas chromatography (GC) coupled to an ISQ LT single quad mass spectrometer (MS) (Thermo Scientific):** The PTV inlet was operated in CT splitless mode and held at 300°C. Separation was achieved on a Rxi-5HT fused silica column (Restek, USA; 30 m, 0.25 mm ID, 0.25µm film) with a He flow rate of 1.5 ml/min using the following temperature program: 2 min hold at 40°C; 25°C/min to 120°C; 6°C/min to 320°C; 30 min hold at 320°C. The ISQ LT was operated in electron ionization mode with a 230°C source temperature, scanning a mass range of 50-500 Da with a 0.2 s dwell time.

**HP 6890 GC coupled to a HP 5973 mass selective detector (Agilent Technologies):** The inlet was operated in splitless mode and held at 320°C. Separation was achieved on a Rxi-5HT fused silica column (Restek, USA; 30 m, 0.25 mm ID, 0.25µm film) with a He flow rate of 2 ml/min using the following temperature program: 1 min hold at 60°C, 7°C/min to 320°C; 10 min hold at 320°C, solvent delay time of 6 min. The HP 5973 MSD was operated in electron ionization mode with a 230°C source temperature, 150°C quad temperature, scanning a mass range of 50-500 Da with a 100 ms dwell time.

**Quantification of compounds**

Standard mixtures of *n*-alkane (C_10_–C_40_) and PAHs (16 priority PAHs) (Sigma-Aldrich) were measured at concentrations ranging from 2.5 to 20 ng. Calibration curves (e.g, Fig 1) created from plotting GCMS peak area vs. real concentrations were used to get the response factor r_x_ (the slope of the fitted linear line) for each of the compound. The concentration (in ng) of compound x in samples were calculated as: A_x_/r_x_

**Figure 1:** An example of a calibration curve for a *n-*alkane compound
