# Supplementary material for: The influence of pressure on crude oil biodegradation in shallow and deep Gulf of Mexico sediments
Source: PLoS One. 2018 Jul 3;13(7):e0199784. doi: 10.1371/journal.pone.0199784 (PMC6029805; doi:10.1371/journal.pone.0199784)
Supplement: S2 Appendix — (DOCX) [file pone.0199784.s002.docx]

**S2 Appendix. Distribution of hopanes and triaromatic sterane compound groups**

Based on Fig S2-1, the variation range of day 18 data in all isomeric ratios of hopane compounds (blue circles) is similar to that of day 0 data (orange circles), suggesting the variations observed were probably within measurement variation. We then performed two tailed t-test to compare the difference between day 18 and day 0 populations with a null hypothesis H_o_ that μ_day 0_  = μ_day 18._ For all ratios, the tests resulted in p values much greater than the significant level α = 0.01, we couldn’t reject H_o_, hence the difference between day 0 and day 18 samples might not be significant (Table S2-1). Similarity in hopane distribution between day 0 and day 18 samples was also shown in the *m/z* 191 trace of hopane chromatograms in Fig S2-2. Similarity in triaromatic sterane (TAS) distribution between day 0 and day 18 samples (*m/z* 231) was demonstrated in Fig S2-3.


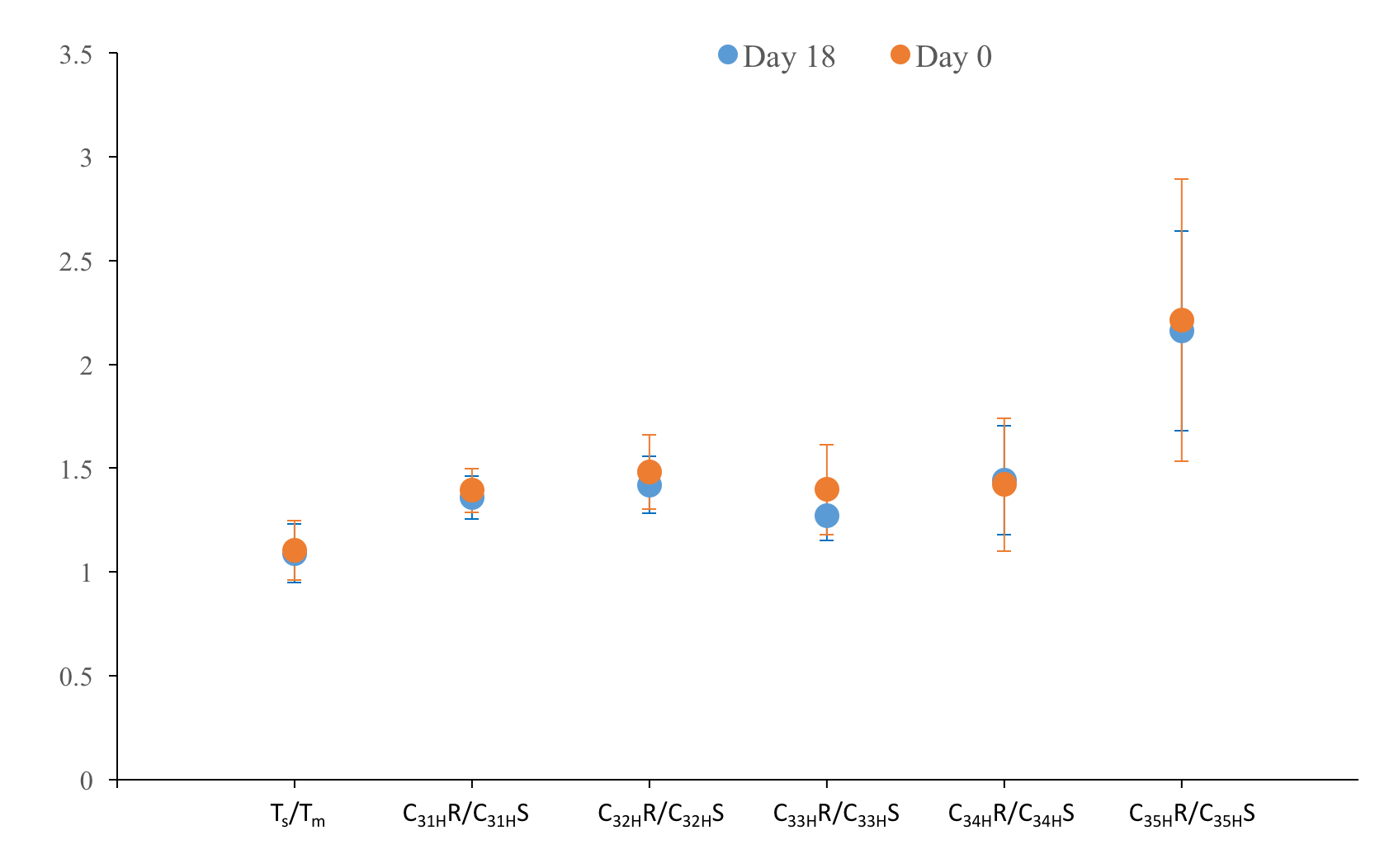


**Figure S2-1:** Ratios of different isomers, including trisnorhopanes (Ts/Tm) and 22R/22S ratios of 17α(H),21β(H) C31-35 homohopanes

**Table S2-1:** Two-tail t test result for different isomer ratio of the hopanes compound groups. The significant level chosen was α = 0.05 and if p < α, we rejected the null hypothesis that **μ_Day 18_ = μ_Day 0_**

| **Ratio** | **Group** | **Mean μ** | **Variance s^2^** | **t** | **p** |
| --- | --- | --- | --- | --- | --- |
| T_s_/T_m_ | Day 18 | 1.10 | 0.02 | 0.32 | 0.75 |
|  | Day 0 | 1.09 | 0.02 |  |  |
| C_31_R/C_31_S | Day 18 | 1.39 | 0.01 | 1.03 | 0.31 |
|  | Day 0 | 1.36 | 0.01 |  |  |
| C_32_R/C_32_S | Day 18 | 1.48 | 0.03 | 1.18 | 0.25 |
|  | Day 0 | 1.42 | 0.02 |  |  |
| C_33_R/C_33_S | Day 18 | 1.4 | 0.05 | 2.11 | 0.05 |
|  | Day 0 | 1.27 | 0.01 |  |  |
| C_34_R/C_34_S | Day 18 | 1.42 | 0.11 | -0.22 | 0.83 |
|  | Day 0 | 1.44 | 0.07 |  |  |
| C_35_R/C_35_S | Day 18 | 2.21 | 0.49 | 0.26 | 0.79 |
|  | Day 0 | 2.16 | 0.24 |  |  |


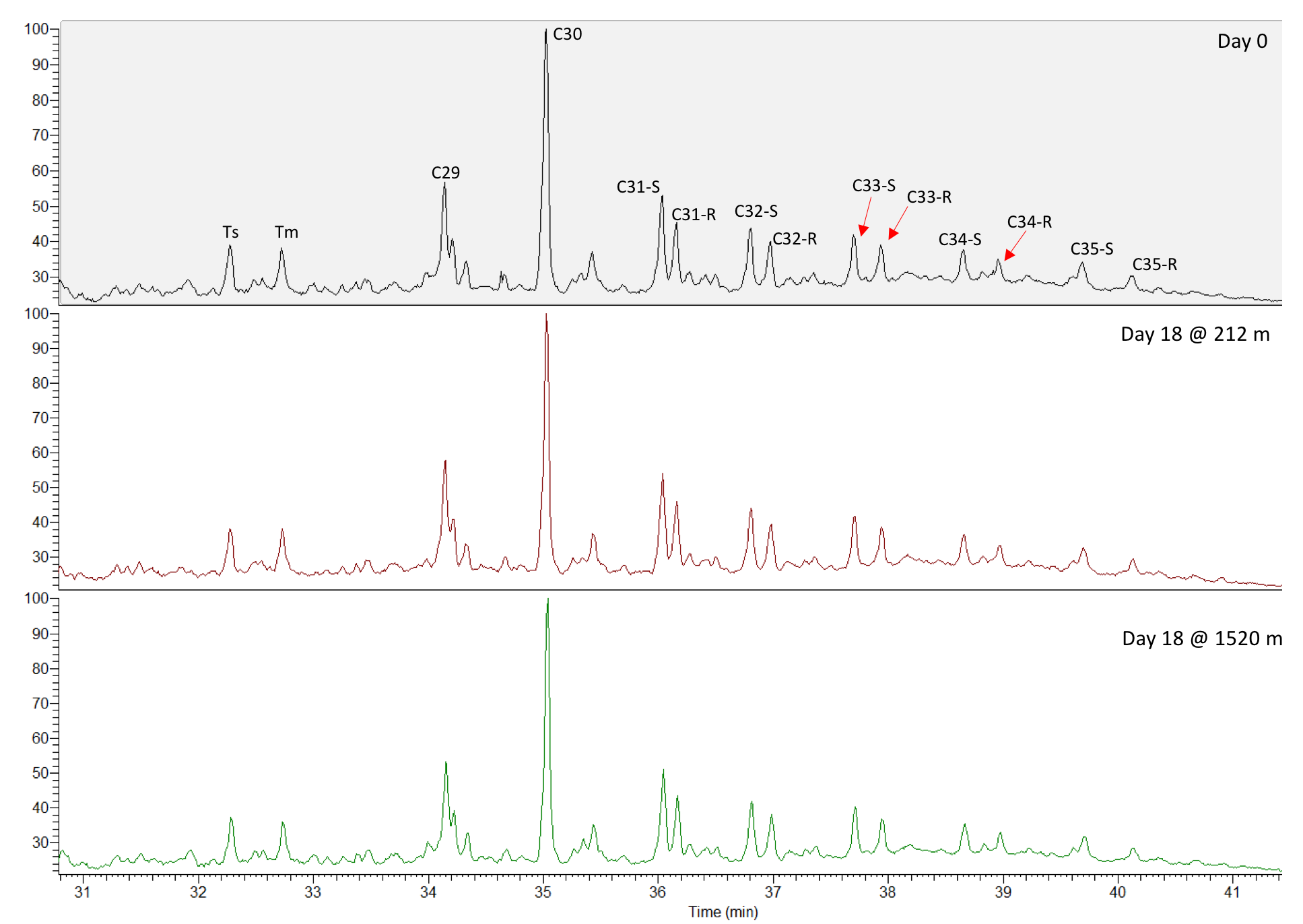


**Figure S2-2:** Example chromatograms of hopanes (*m/z* 191) distribution in day 0 and day 18 samples.


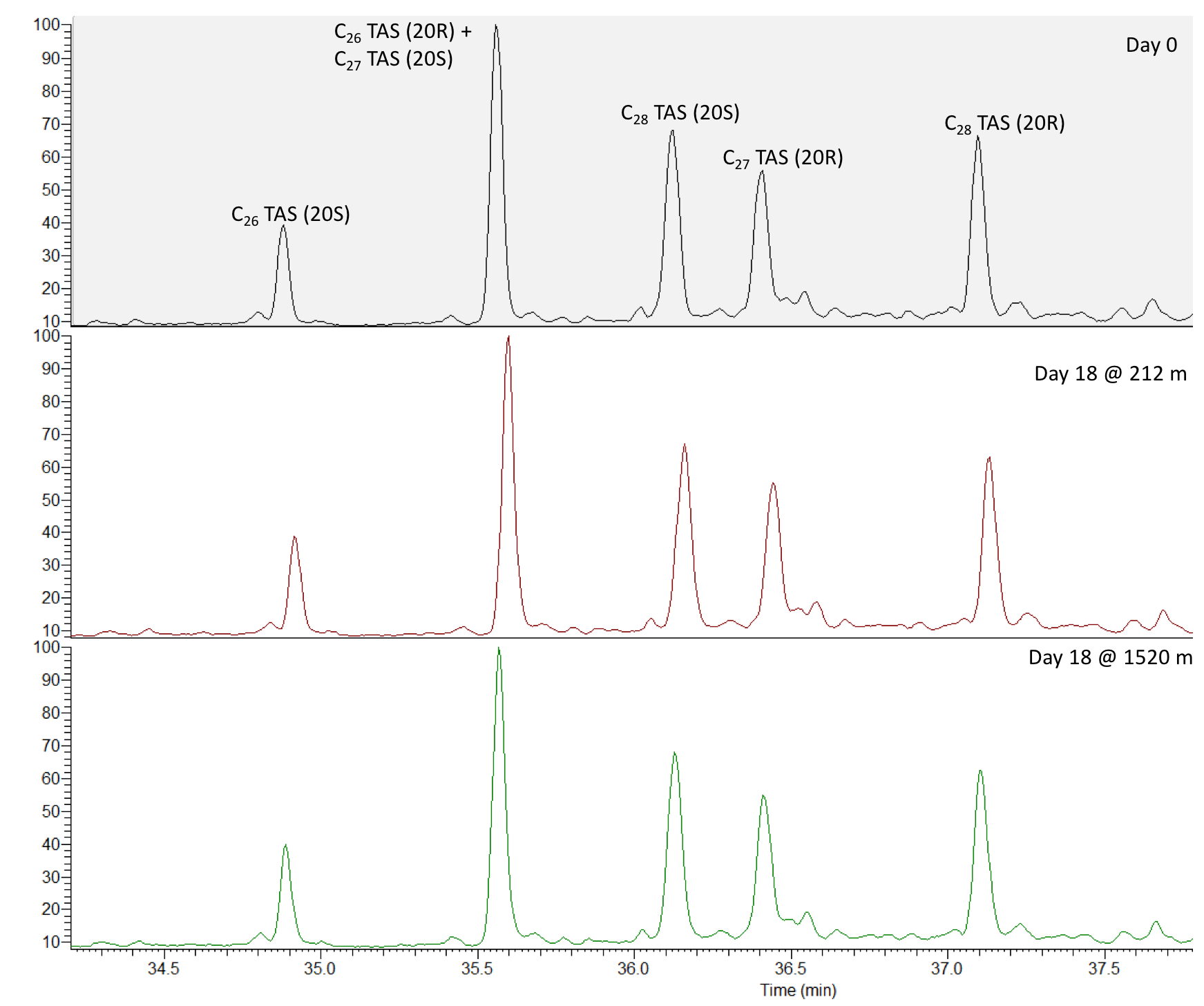


**Figure S2-3:** Examples of triaromatic steranes (*m/z* 231) distribution in day 0 and day 18 samples
