# Supplementary material for: The influence of pressure on crude oil biodegradation in shallow and deep Gulf of Mexico sediments
Source: PLoS One. 2018 Jul 3;13(7):e0199784. doi: 10.1371/journal.pone.0199784 (PMC6029805; doi:10.1371/journal.pone.0199784)
Supplement: S3 Appendix — (DOCX) [file pone.0199784.s003.docx]

**S3 Appendix. Calculation of n-alkane depletion due to off-gassing at 15 Mpa**

If we considered all PAHs loss at 15 MPa (~35%) was due to off-gassing, we back calculated how much off-gassing might affect the loss in total n-alkanes (~ 42%) at 15 Mpa. Vapor pressure (VP) (EPI suite,^5^ at 25^0^C) ranged from 1E-3 to 1.6E-9 mm Hg for PAHs we analyzed and ranged from 1.5E-2 to 6E-10 mm Hg for C15-30 n-alkanes. Since the number of high-VP PAHs is larger than that of n-alkanes, the amount of n-alkanes lost through off-gassing should be less than that of PAHs. Since total n-alkane (C_15-40_) was ~ 10 time greater than total PAH, off-gassing should only account for a maximum of ~ 3.5% loss in total n-alkanes.

**Table S3-1:** Vapor pressures (mm Hg, at 25^0^C) of analyzed n-alkanes (A) and PAHs (B), using EPI suite (US, EPA).^5^ Key: PNT = phenanthrene, F = fluorine, DBT = dibenzothiophene, P = pyrene, Chy = chrysene; M = monomethyl, E = ethyl, DM = dimethyl, TM = trimethyl

**A**

**B**

| Compound | *Vapor pressure*  *(mm Hg, 25^0^C)* |
| --- | --- |
| *C15* | 1.53E-02 |
| *C16* | 6.96E-03 |
| *C17* | 3.24E-03 |
| *C18* | 1.46E-03 |
| *C19* | 6.73E-04 |
| *C20* | 3.10E-04 |
| *C21* | 1.42E-04 |
| *C22* | 6.92E-05 |
| *C23* | 3.54E-05 |
| *C24* | 1.69E-05 |
| *C25* | 9.63E-06 |
| *C26* | 5.26E-06 |
| *C27* | 9.84E-07 |
| *C28* | 1.55E-06 |
| *C29* | 9.56E-07 |
| *C30* | 5.62E-07 |
| *C31* | 3.41E-07 |
| *C32* | 2.00E-07 |
| *C33* | 6.94E-08 |
| *C34* | 7.76E-08 |
| *C35* | 4.99E-08 |
| *C36* | 9.05E-09 |
| *C37* | 6.09E-09 |
| *C38* | 3.89E-10 |
| *C39* | 3.30E-11 |
| *C40* | 5.98E-10 |

| Compound | *Vapor pressure*  *(mm Hg, 25^0^C)* |
| --- | --- |
| *PNT* | 4.32E-05 |
| *1-MPNT* | 2.45E-05 |
| *2-MPNT/3-MPNT* | 6.67E-05 |
| *9-MPNT* | 5.01E-05 |
| *2-EPNT* | 2.12E-05 |
| *9-EPNT* | 5.43E-05 |
| *9,10-DMPNT* | 7.59E-06 |
| *2,5-DMPNT* | 1.82E-05 |
| *3,6-DMPNT* | 7.59E-06 |
| *1,7/2,3/3,5-DMPNT* | 1.82E-05 |
| *2,3,5-TMPNT* | 8.20E-06 |
| *1E-2M-PNT* | 7.73E-06 |
| *DBT* | 1.65E-04 |
| *3-MDBT* | 7.00E-05 |
| *1,2/1,3/2,3-DMDBT* | 2.75E-05 |
| *EthylDBT* | 2.97E-05 |
| *4E-6M-DBT* | 1.20E-05 |
| *TrimethylDBT* | 1.11E-05 |
| *1-MF* | 3.00E-04 |
| *2-MF/3-MF* | 3.98E-04 |
| *9-MF* | 1.05E-03 |
| *2-EF* | 8.22E-04 |
| *2,3-DMF* | 7.68E-04 |
| *9-EF* | 2.34E-04 |
| *1,9-DMF* | 2.02E-04 |
| *9,9-DMF* | 3.28E-04 |
| *Pyrene* | 3.44E-07 |
| *MP* | 1.75E-06 |
| *DMP* | 8.00E-07 |
| *TMP* | 3.15E-07 |
| *Chrysene* | 1.56E-09 |
| *5-MChy* | 5.45E-07 |
| *2-MChy & 3-MChy* | 2.53E-07 |
| *1-MChy* | 1.35E-08 |
| *DMChy* | 1.14E-07 |
| *TMChy* | 4.44E-08 |

**Figure S3-1:** Counts of compounds belong to n-alkanes and PAHs, classified to different ranges of vapor pressure (VP) from 10^-2^(E-2) to 10^-11^(E-11) mm Hg at 25^0^C. For instance, E-2 means 10^-2^ < VP < 10^-3^mm Hg and so on.
