# Supplementary material for: The influence of pressure on crude oil biodegradation in shallow and deep Gulf of Mexico sediments
Source: PLoS One. 2018 Jul 3;13(7):e0199784. doi: 10.1371/journal.pone.0199784 (PMC6029805; doi:10.1371/journal.pone.0199784)
Supplement: S4 Appendix — (DOCX) [file pone.0199784.s004.docx]

**S4 Appendix. Background hydrocarbons in un-incubated sediments**

Peak areas of quantitative ions m/z 57 and 191 were used to quantify total n-alkanes and C_30_-hopane respectively. Area was approximated to weight, using internal standard d10-phenanthrene. Concentrations were reported as µg n-alkanes and ng hopane per gram of dry sediments (table S.5).

**Table S4-1:** Background hydrocarbons of the surface sediments (0-4mm) at different water depths, measured as total n-alkanes (µg/g sediment) and C_30_ hopane (ng/g sediment)

| **site** | **water depth (m)** | **Total n-alkanes (µg/g sediment)** | **C_30_ hopane**  **(ng/g sediment)** |
| --- | --- | --- | --- |
| SL1240 | 62 | 1.50 | 7.34 |
| PCB03 | 96 | 0.15 | 1.19 |
| SL980 | 150 | 0.88 | 4.21 |
| SL7150 | 196 | 3.63 | 0.00 |
| SL1460 | 212 | 2.47 | 16.74 |
| SL8100 | 226 | 0.57 | 1.97 |
| SL9150 | 251 | 0.71 | 4.87 |
| MC04 | 399 | 1.24 | 1.81 |
| MC06 | 595 | 0.88 | 3.31 |
| PCB09 | 981 | 1.07 | 2.04 |
| PCB06 | 1008 | 0.82 | 10.52 |
| DSH08 | 1127 | 1.33 | 14.45 |
| DSH10 | 1520 | 0.66 | 3.64 |
