# Supplementary material for: The influence of pressure on crude oil biodegradation in shallow and deep Gulf of Mexico sediments
Source: PLoS One. 2018 Jul 3;13(7):e0199784. doi: 10.1371/journal.pone.0199784 (PMC6029805; doi:10.1371/journal.pone.0199784)
Supplement: S5 Appendix — (DOCX) [file pone.0199784.s005.docx]

**S5 Appendix. Calculating contamination level**

In our incubations, we used 5 µL oil, equivalent to 4 mg oil (Macondo density 0.82 g/cm^3^).^6^ Using the concentration of C_30_-hopane in Macondo oil (58 µg/g) measured by Valentine et al. (2014) and an average of sediment dry weight of 0.2g that was used in each incubation, we calculated an average concentration of ~ 1.1 µg C_30_-hopane/g sediment in our experiments.

**Reference**

Valentine D.L., Fisher G.B., Bagby S.C., Nelson R.K., Reddy, C.M., Sylva, S.P., et al. Fallout plume of submerged oil from Deepwater Horizon. Proc Natl Acad Sci. 2014**,** 111(45): 15906–1591.
