# Supplementary material for: The influence of pressure on crude oil biodegradation in shallow and deep Gulf of Mexico sediments
Source: PLoS One. 2018 Jul 3;13(7):e0199784. doi: 10.1371/journal.pone.0199784 (PMC6029805; doi:10.1371/journal.pone.0199784)
Supplement: S1 Table — (DOCX) [file pone.0199784.s006.docx]

| **Fraction** | **Compound** | **Quantitative ion m/z** |
| --- | --- | --- |
| **Aliphatics** | C_15_ – C_40_ n-alkanes | 57 |
|  | Pristane | 57 |
|  | Phytane | 57 |
|  | Cyclohexane and branched cyclohexanes | 83 |
|  | C_29_ – C_35_ homohopanes (including R and S isomers) | 191 |
|  | C_29_ sterane | 217 |
| **Aromatics** | C_4_ Naphthalene | 184 |
|  | Phenanthrene | 178 |
|  | C_1_ Phenanthrene | 192 |
|  | C_2_ Phenanthrene | 206 |
|  | C_3_ Phenanthrene | 220 |
|  | C_4_ Phenanthrene | 234 |
|  | Dibenzothiophene | 184 |
|  | C_1_ Dibenzothiophene | 198 |
|  | C_2_ Dibenzothiophene | 212 |
|  | C_3_ Dibenzothiophene | 226 |
|  | C_1_ Fluorene | 180 |
|  | C_2_ Fluorene | 194 |
|  | C3 Fluorene | 208 |
|  | C_1_ Pyrene | 216 |
|  | C_2_ Pyrene | 230 |
|  | C_3_ Pyrene | 244 |
|  | Chrysene | 228 |
|  | C_1_ Chrysene | 242 |
|  | C_2_ Chrysene | 256 |
|  | C_3_ Chrysene | 270 |
|  | C_26_-Triaromatic sterane | 231 |
