# Supplementary material for: The influence of pressure on crude oil biodegradation in shallow and deep Gulf of Mexico sediments
Source: PLoS One. 2018 Jul 3;13(7):e0199784. doi: 10.1371/journal.pone.0199784 (PMC6029805; doi:10.1371/journal.pone.0199784)
Supplement: S2 Table — Each compound group is identified based on a quantitative ion and a confirmation ion m/z (Zeigler et al., 2008; Robbat Jr. and Wilton, 2014*). * Zeigler C., MacNamara K., Wang Z., Robbat Jr. A. Total alkylated polycyclic aromatic hydrocarbon characterization and quantitative comparison of selected ion monitoring versus full scan gas chromatography/mass spectrometry based on spectral deconvolution. Journal of Chromatography A 2008; 1205, 109–116. Robbat Jr., A.; Wilton, N.M. A new spectral deconvolution–Selected ion monitoring method for the analysis of alkylated polycyclic aromatic hydrocarbons in complex mixtures. Talanta 2014 125, 114–124. (DOCX) [file pone.0199784.s007.docx]

| **Group number** | **Starting time (mins)** | **PAHs (quantitative and confirmation m/z ions)** |
| --- | --- | --- |
| 1 | 6 | C_1_ Napthalene 141, 142  C_2_ Napthalene 156, 141  C_3_ Napthalene 170, 155 |
| 2 | 18.14 | C_4_ Napthalene 184, 169  C_1_ Fluorene 180, 165  Dibenzothiophene 184 |
| 3 | 21.25 | C_2_ Fluorene 194, 179  C_3_ Fluorene 208, 165  Phenanthrene 178, 176  C_1_ Phenanthrene 192, 191  C_2_ Phenanthrene 206, 191  C_1_ Dibenzothiophene 198, 197  C_2_ Dibenzothiophene 212, 197 |
| 4 | 24.45 | C_3_ Dibenzothiophene 226, 211  C_3_ Phenanthrene 220, 205  Pyrene 202, 203 |
| 5 | 27.61 | C_3_ Phenanthrene 220, 205  C_4_ Phenanthrene 234, 219  C_1_ Pyrene 216, 215  C_2_ Pyrene 230, 229  C_3_ Pyrene 244, 229  Chrysene 228, 226  C_1_ Chrysene 242, 241 |
| 6 | 32.30 | C_2_ Chrysene 256, 241  C_3_ Chrysene 270, 239  Triaromatic sterane 231 |
