# Supplementary material for: The influence of pressure on crude oil biodegradation in shallow and deep Gulf of Mexico sediments
Source: PLoS One. 2018 Jul 3;13(7):e0199784. doi: 10.1371/journal.pone.0199784 (PMC6029805; doi:10.1371/journal.pone.0199784)
Supplement: S3 Table — (TOC: total organic carbon, Carb: carbonate in the sediments, P: pressure, T: temperature, WD: water depth, SED: sediment fraction, WAF: water fraction). (DOCX) [file pone.0199784.s008.docx]

| **ID** | **Site** | **TOC**  **(wt %)** | **Carb**  **(wt %)** | **P**  **(MPa)** | **T (^o^C)** | **WD**  **(m)** | ***n*-alkane SED (%)** | ***n*-alkane**  **WAF (%)** | **PAH**  **SED (%)** | **PAH**  **WAF (%)** |
| --- | --- | --- | --- | --- | --- | --- | --- | --- | --- | --- |
| 1 | DSH08 | 1.96 | 26.1 | 11.1 | 4 | 1127 | 60.54 | 32.19 | 39.09 | 22.93 |
| 2 | MC04 | 2.06 | 50.9 | 4 | 10 | 399 | 83.14 | 56.66 | 9.41 | 18.80 |
| 2 | MC04 | 2.06 | 50.9 | 4 | 10 | 399 | 88.46 | 55.87 | 11.04 | 24.10 |
| 3 | PCB03 | 0.84 | 78.1 | 0.1 | 20 | 96 | 90.44 | 97.60 | 15.30 | 19.12 |
| 3 | PCB03 | 0.84 | 78.1 | 0.1 | 20 | 96 | 92.63 | 92.69 | 30.02 | 16.45 |
| 4 | SL1460 | 1.43 | 43.8 | 2.5 | 20 | 212 | 98.00 | 82.83 | 60.06 | 35.81 |
| 5 | SL1240 | 1.73 | 9.5 | 0.1 | 20 | 62 | 80.96 | 49.30 | 11.81 | 22.09 |
| 5 | SL1240 | 1.73 | 9.5 | 0.1 | 20 | 62 | 89.03 | 58.34 | 8.95 | 40.35 |
| 6 | PCB09 | 1.58 | 40.8 | 10.5 | 4 | 981 | 61.03 | 47.60 | 22.44 | 16.29 |
| 6 | PCB09 | 1.58 | 40.8 | 10.5 | 4 | 981 | 65.43 | 56.02 | 27.15 | 24.14 |
| 7 | SL980 | 2.29 | 29.6 | 2.5 | 20 | 150 | 96.57 | 87.50 | 84.31 | 62.22 |
| 7 | SL980 | 2.29 | 29.6 | 2.5 | 20 | 150 | 96.63 | 74.44 | 74.97 | 60.44 |
| 8 | SL9150 | 2.21 | 32.1 | 2.5 | 10 | 251 | 95.58 | 82.25 | 54.52 | 33.62 |
| 8 | SL9150 | 2.21 | 32.1 | 2.5 | 10 | 251 | 94.73 | 75.65 | 51.08 | 28.64 |
| 9 | SL7150 | 1.49 | 69.9 | 1.9 | 10 | 196 | 93.55 | 72.42 | 18.32 | 21.43 |
| 9 | SL7150 | 1.49 | 69.9 | 1.9 | 10 | 196 | 92.54 | 64.27 | 23.76 | 12.40 |
| 10 | PCB06 | 1.71 | 31 | 9.4 | 4 | 1008 | 59.11 | 40.95 | 28.77 | 17.27 |
| 10 | PCB06 | 1.71 | 31 | 9.4 | 4 | 1008 | 56.60 | 46.11 | 22.49 | 24.83 |
| 11 | SL8100 | 2.23 | 56.7 | 1.9 | 10 | 226 | 85.08 | 69.27 | 23.80 | 40.19 |
| 11 | SL8100 | 2.23 | 56.7 | 1.9 | 10 | 226 | 93.53 | 85.18 | 25.77 | 10.75 |
| 12 | MC06 | 1.70 | 44.2 | 5.8 | 4 | 595 | 84.95 | 65.82 | 19.66 | 29.25 |
| 12 | MC06 | 1.70 | 44.2 | 5.8 | 4 | 595 | 78.23 | 72.34 | 15.73 | 44.31 |
| 13 | DSH10 | 1.55 | 20.3 | 15.28 | 4 | 1520 | 49.54 | 32.00 | 67.33 | 37.32 |
| 13 | DSH10 | 1.55 | 20.3 | 15.28 | 4 | 1520 | 46.01 | 17.39 | 67.92 | 30.01 |
