# Supplementary figures and images for: The influence of pressure on crude oil biodegradation in shallow and deep Gulf of Mexico sediments
Source: PLoS One. 2018 Jul 3;13(7):e0199784. doi: 10.1371/journal.pone.0199784 (PMC6029805; doi:10.1371/journal.pone.0199784)

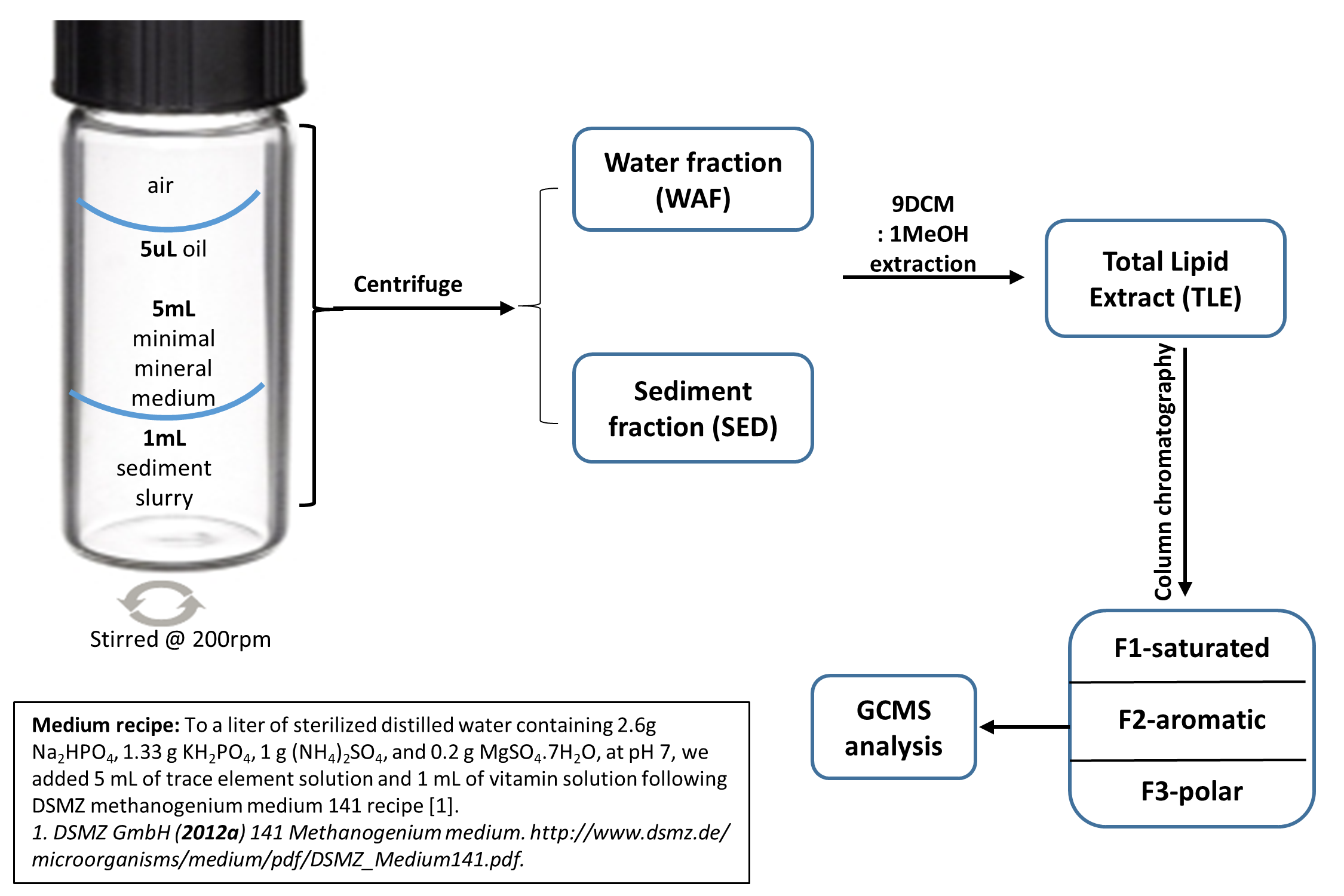

Supplement: S1 Fig — (TIF) [file pone.0199784.s009.tif]

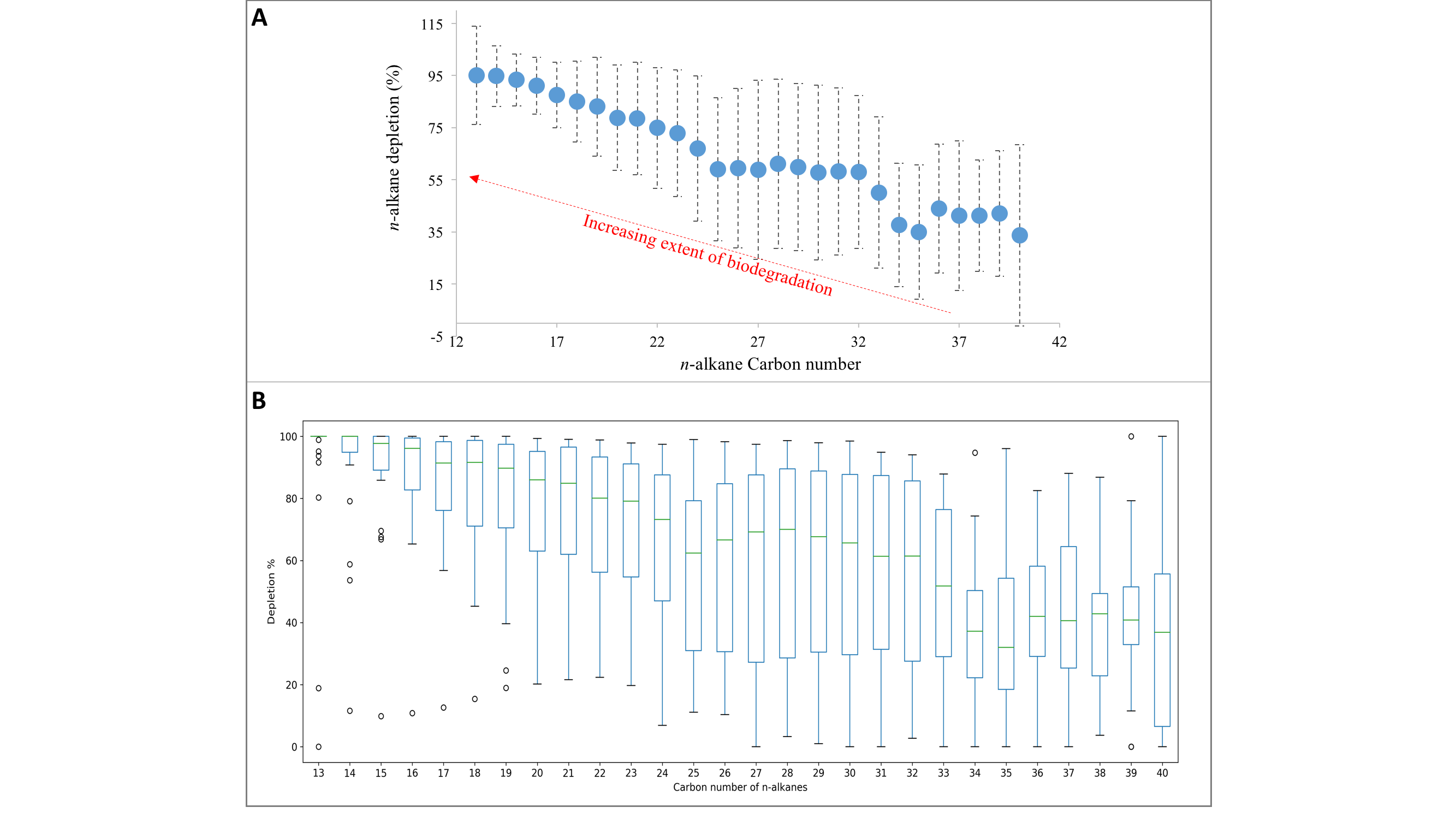

Supplement: S2 Fig — A. Mean and standard errors plot for depletion of each n-alkane for all day-18 samples; B. Boxplot for depletion of each n-alkane for all day-18 samples. (TIFF) [file pone.0199784.s010.tiff]

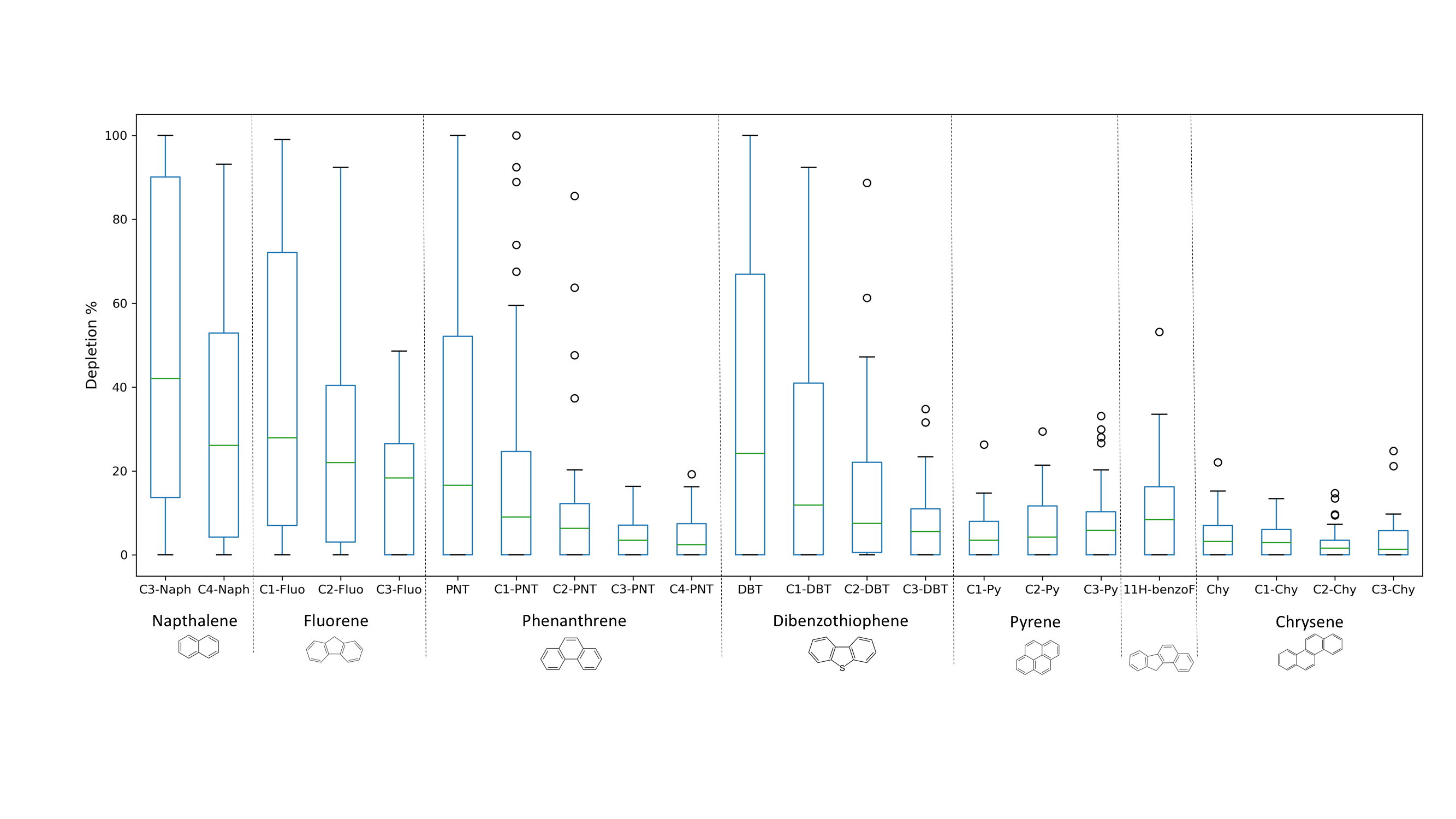

Supplement: S3 Fig — (C1: methyl, C2: ethyl or dimethyl, C3: trimethyl, C4: tetramethyl; Naph: napthalene, PNT: phenanthrene, Fluo: fluorene, DBT: dibenzothiophene, Py: pyrene, 11H-benzoF: 11H-benzo[b]fluorene, Chy: chrysene). (TIFF) [file pone.0199784.s011.tiff]

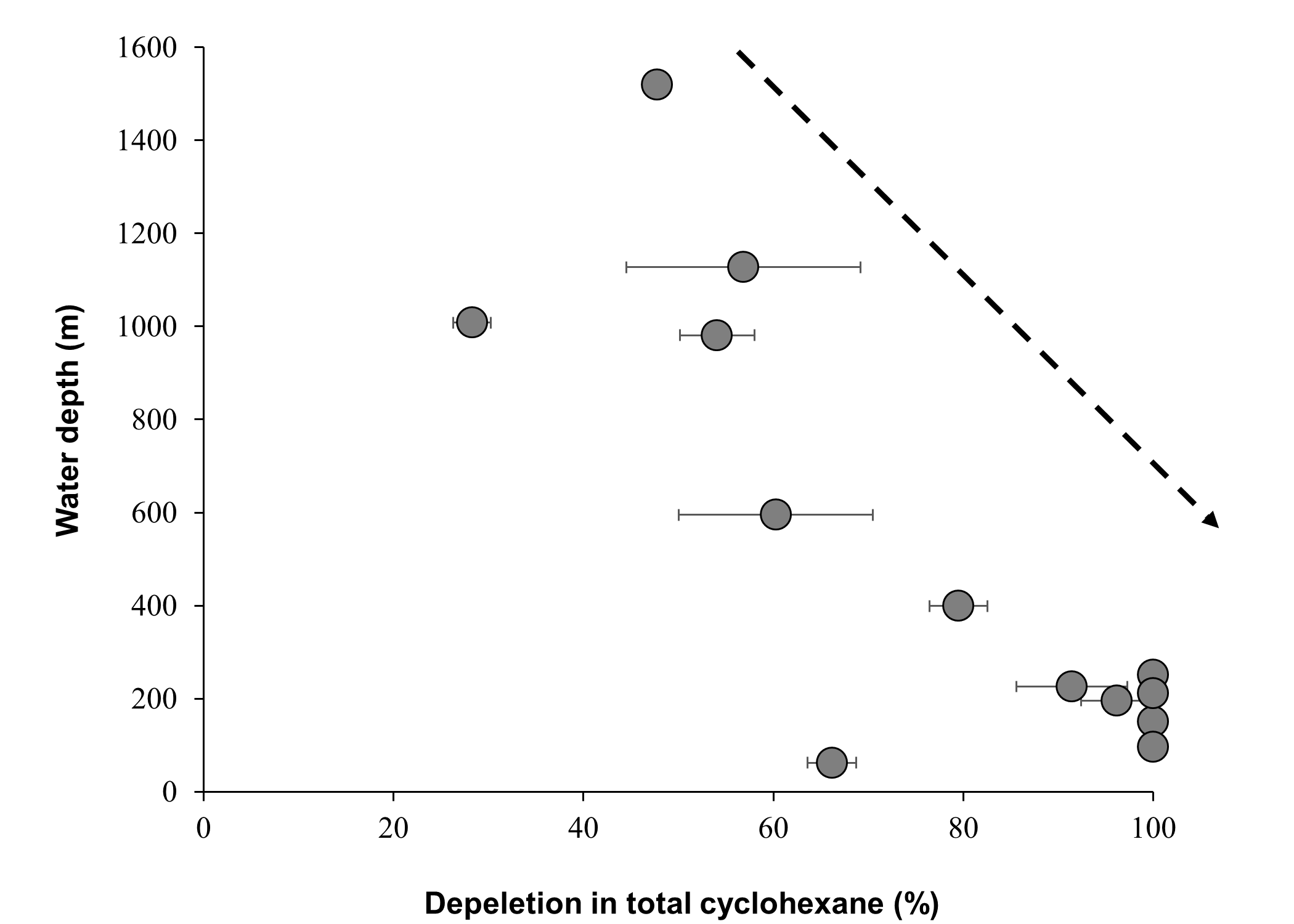

Supplement: S4 Fig — The dashed arrow represents interpreted direction of increasing biodegradation extent. (TIF) [file pone.0199784.s012.tif]

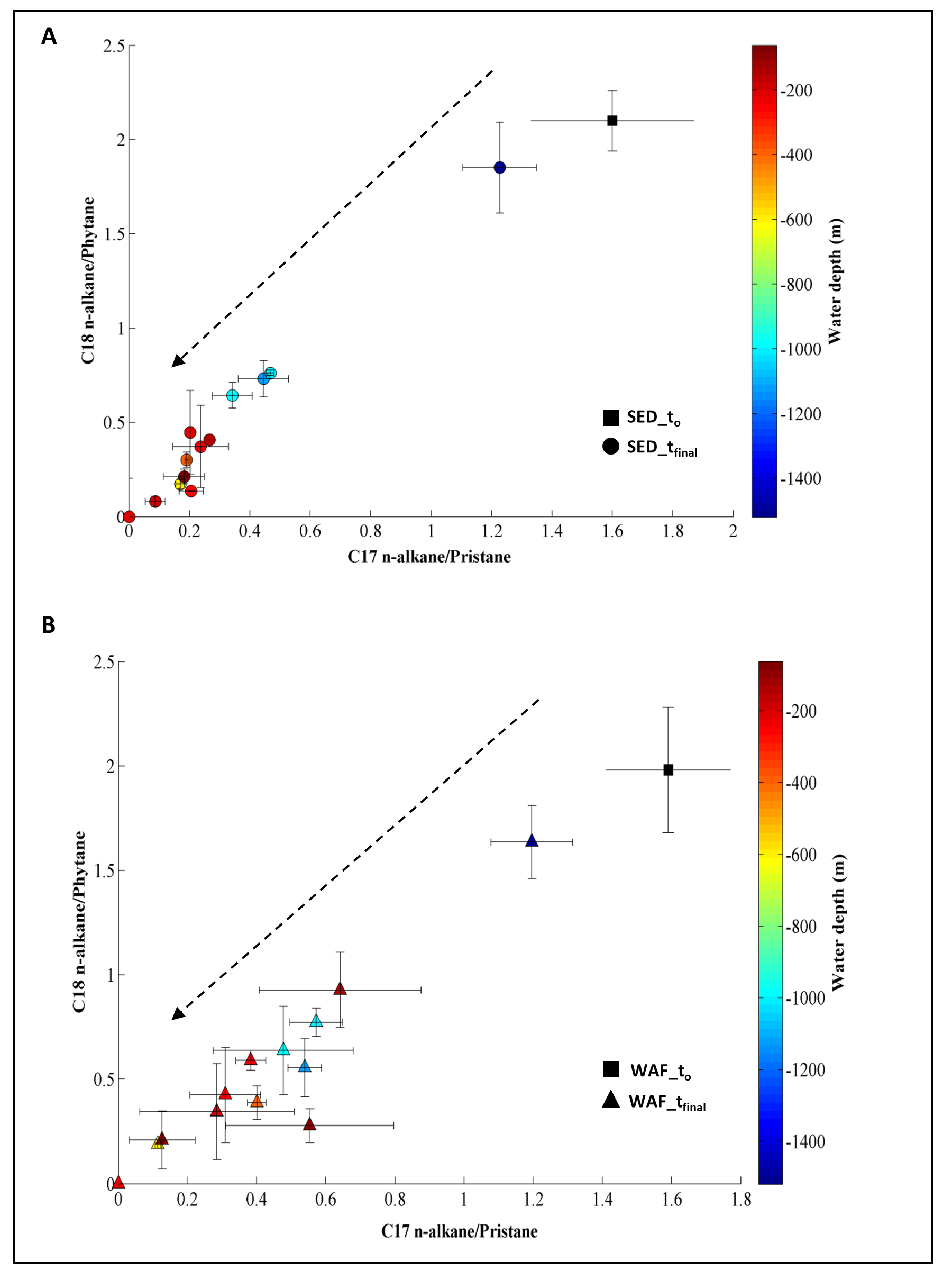

Supplement: S5 Fig — A, sediment fractions (SED) and B, water fractions (WAF). Initial ratios are represented by the black square. Samples are color-coded according to sampling water depths. The dashed arrow represents interpreted direction of increasing biodegradation extent. (TIF) [file pone.0199784.s013.tif]

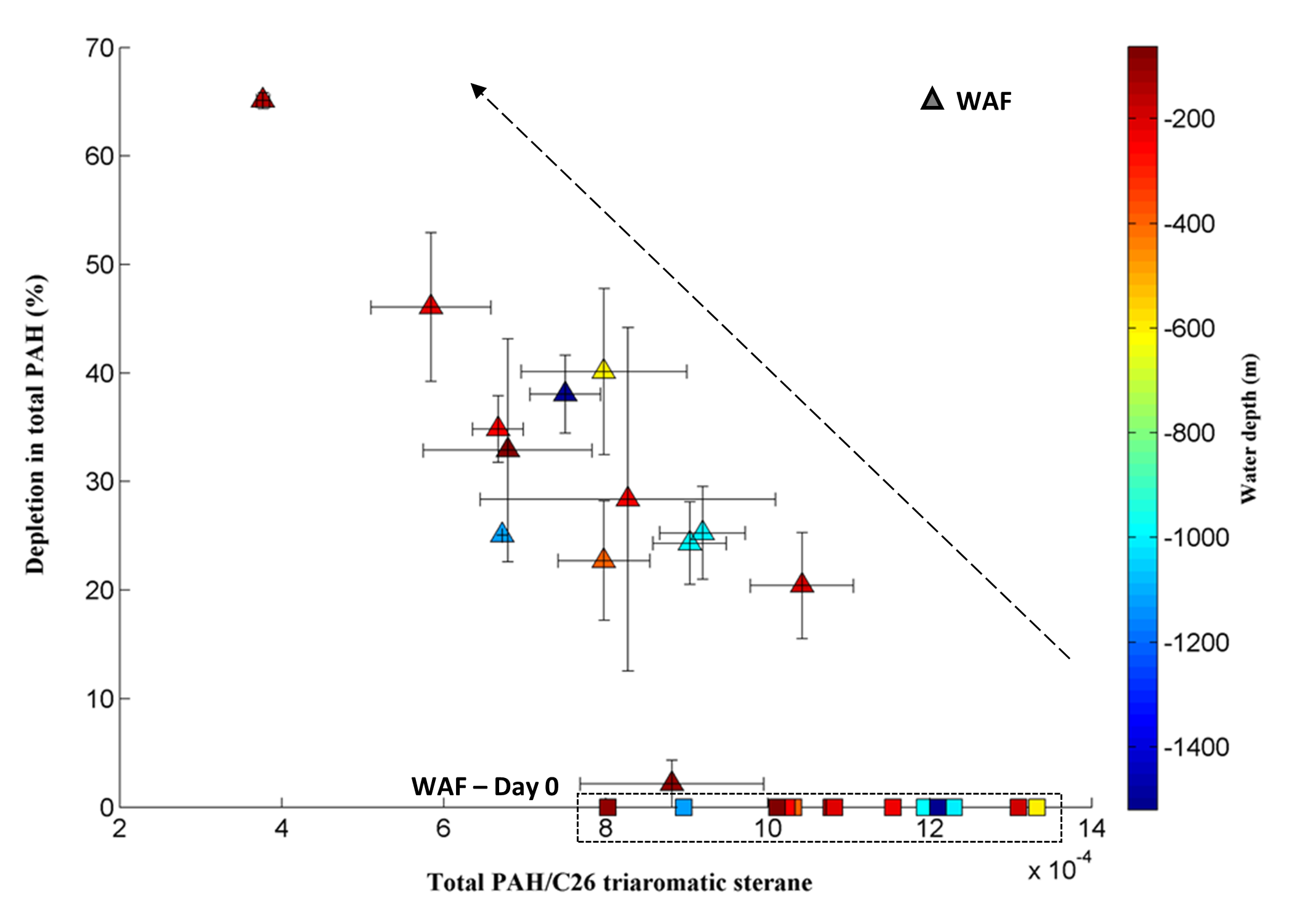

Supplement: S6 Fig — Initial total PAHs are represented by squares. Samples are color-coded according to sampling water depths. The dashed arrow represents interpreted direction of increasing biodegradation extent. (TIF) [file pone.0199784.s014.tif]
